# The dilemma of valuing geodiversity: geoconservation versus geotourism

**DOI:** 10.1098/rsta.2023.0049

**Published:** 2024-04-01

**Authors:** S. Anougmar, A. Meesters, D. van Ree, T. Compernolle

**Affiliations:** ^1^ Department of Engineering Management, University of Antwerp, Prinsstraat 13, 2000 Antwerp, Belgium; ^2^ Vrije Universiteit Amsterdam, Institute for Environmental Studies, De Boelelaan 1087, 1081 HV, Amsterdam, The Netherlands; ^3^ Deltares, Boussinesqweg 1, 2629 HV, Delft, The Netherlands; ^4^ Geological Survey of Belgium, Royal Belgian Institute of Natural Sciences, Jennerstraat 13, 1000 Brussels, Belgium

**Keywords:** geodiversity, geosystem services, economic valuation, systematic literature review

## Abstract

Geodiversity and geosystem services are confronting global threats. However, the majority of conservation strategies tend to overlook the geological component within ecosystems. The existing literature centres on biodiversity, ecosystem services and their economic valuation. In this paper, we conduct a systematic literature review to identify the gap in the assessment of geological diversity, pinpointing areas where scientific contributions are needed to safeguard geological resources. Our findings reveal a concentration of studies assessing geodiversity in European and Asian countries. While the majority of the reviewed papers emphasizes the recreational features and associated values of geological resources, promoting geotourism and recognizing its potential for economic growth, there is a significant oversight concerning the impact of tourism on geological resources. Existing assessments predominantly focus on visitors' perceptions and preferences, sidelining the inhabitants’ perspective and their crucial roles in the conservation of geodiversity.

This article is part of the Theo Murphy meeting issue ‘Geodiversity for science and society’.

## 1. Introduction

Geodiversity and geosystem services represent relatively recent concepts in the literature. The term ‘geodiversity' was first introduced in 1993 following the Rio Earth Summit of 1992 [1]. It is defined as the inherent diversity within geological (rocks, minerals, fossils), geomorphological (landforms, topography, physical processes), soil and hydrological features; it encompasses their assemblages, structures, systems and contributions to landscapes [2].

Geodiversity is essentially regarded as the non-living counterpart to biodiversity, signifying the diverse range of abiotic elements present in ecosystems. Similarly, geosystem services, also known as abiotic ecosystem services, encompass the goods and functions derived from geodiversity. This analogy draws parallels with ecosystem services, where biodiversity defines the goods and functions originating from living components [2,3]. The concept of geosystem services was developed to support the sustainable development of the subsurface [4,5]. However, it is noted that many geodiversity papers still lack integration with the geosystem services framework.

### (a) Including geodiversity and geosystem services in decision-making

Currently, most research studies focus on the economic value of biodiversity and ecosystem services in national parks (e.g. [6,–8]). However, only a few studies assess the economic value of geodiversity and geosystem services [2]. Frameworks and literature on ecosystem services rarely consider geodiversity and geosystem services [5]. Van der Meulen *et al*. [9] further highlight the bias within the existing ecosystem services classifications emphasizing the biotic aspects of ecosystems. It is essential to recognize that ecosystems result from biotic and abiotic structures and processes. Hence, including abiotic services in the classification systems would significantly contribute to effective planning and decision-making. The term ‘ecosystem services approach’ refers to the methods mostly used to value the natural environment and ecosystems. The Millennium Ecosystem Assessment (MEA), the Economics of Ecosystems and Biodiversity (TEEB) and the Common International Classification of Ecosystem Services (CICES) are three main classification systems commonly employed by decision-makers to assess ecosystem services and biodiversity. All three of these classification systems are designed only to consider the biotic side of biodiversity, and the abiotic side is often overlooked [10]. Societies rely extensively on both ecosystem and geosystem services, encompassing both biotic and abiotic processes. Therefore, when formulating decisions for ecosystem management, it is imperative to adopt a balanced approach that takes into account both the living and non-living components of nature including the subsurface [2,11,12]. The diverse geological, geomorphological and soil characteristics and processes on Earth play a fundamental role in the environment. They contribute significantly to vital functions such as water purification and erosion control [13]. However, the underrepresentation of geosystem services in scientific literature, as noted by Van Ree *et al*. [14], poses a significant challenge. This deficiency can adversely impact decision-making processes in spatial planning, environmental policy and the long-term management of ecosystems. With increasing threats from human activities and climate change on ecosystems, it becomes imperative to adopt an inclusive approach to valuing ecosystems that incorporates both geodiversity and biodiversity. Neglecting either dimension could lead to the undervaluation of nature due to perceived differences, such as the intrinsic value of nature compared to the economic value of minerals. This underscores the need for a comprehensive and integrated perspective in our efforts towards sustainable environmental management [15,16].

The oversight of geodiversity in ecosystem assessments can be attributed, in part, to the distinctive time scale necessary for the replenishment of its resources. Geodiversity, including its abiotic components like minerals and fossil fuels, are non-living resources, diverging from the biotic components of ecosystems. Consequently, the regeneration of geodiversity requires more prolonged periods compared to the more rapidly renewing biotic elements. Furthermore, geodiversity and geosystem goods and services relate in a significant part to the subsurface and are thus invisible to the naked eye.

The replenishment of geodiversity resources unfolds over a geological time scale, spanning thousands to tens of millions of years and more. However, the potential for depletion or degradation occurs on a human time scale, typically compressed within a range of 1–100 years. This temporal incongruity underscores the need to refocus attention on the conservation of geosystem services. Addressing this challenge involves navigating the temporal replenishment differences between ecosystem and geosystem services, as elucidated [9]. Recognizing and mitigating these temporal disparities are paramount for effective management, ensuring the sustainable utilization and preservation of both ecosystem and geosystem services. Geodiversity also is not a static phenomenon as forces of nature result in a continuous change over geological time scales. Some phenomena are relatively fast (e.g. formation of salt domes), other are very slow (e.g. continental drift). Population growth, urbanization and increased standards of living result in rapid changes including rising threats to current geodiversity at much shorter time scales.

### (b) Threats to geodiversity and problems in the management of Geoparks and geological areas

Current threats to geodiversity encompass a range of human-induced and environmental factors, including mineral extraction, urban expansion, land development, coastal and river engineering, forestry activities, vegetation growth, agriculture, recreation and tourism. The impact of these anthropogenic practices on geodiversity is multifaceted, involving soil erosion and destruction, disruption of geomorphological processes, landform damage, disruption of coastal and fluvial processes. Recreational activities such as geotourism further compound the challenges by contributing to fragmentation of site integrity, footpath erosion, and other localized soil erosion and loss of soil organic matter [16]. Beyond human activities, geodiversity faces additional threats from climate change and extreme weather events. A recent study underscores the vulnerability of geodiversity, emphasizing flooding and funding shortages as top threats to designated UNESCO sites in the UK and Canada [17].

To support the conservation and management of geodiversity, UNESCO introduced the ‘Global Geopark' label in 2004. According to UNESCO [18], a UNESCO Global Geopark is defined as ‘a single, unified geographical area where sites and landscapes of international geological significance are managed with a holistic concept of protection, education and sustainable development. It comprises several geological heritage sites of special scientific importance, rarity or beauty, and these features are representative of a region's geological history and the events and processes that formed it'. To ensure the continued operation of Geoparks, legislation for protecting geodiversity becomes necessary [19]. However, this poses a challenge, as the UNESCO label does not classify Geoparks as a category of protected areas. Consequently, Geoparks must use their national legal frameworks to safeguard their geoheritage [20]. Geoheritage refers to scientifically significant geological features, sites and landscapes that hold important information about Earth's history, processes and evolution [1,10].

The management of Geoparks poses various challenges to decision-makers, particularly in navigating the delicate balance between the benefits of geotourism and the conservation of geoheritage. Geotourism, defined as ‘tourism and recreation based on geology and landscapes' [19], emerges as a potential economic catalyst for various regions globally. It has the capacity to create new jobs and stimulate social and economic growth, especially for remote and economically disadvantaged areas possessing rich geological features. However, geotourism poses a real threat to geoheritage [15,19,21,22]. UNESCO identifies geotourism as one of the primary threats to geodiversity when not well managed. Financial constraints add to the challenges, as a lack of resources hinders the effective management and conservation of geological areas [17].

According to Ólafsdóttir & Tverijonaite [21], managing geotourism can be a real challenge in Geoparks and other geological areas. The potential for overcrowding and its subsequent environmental impacts emerges as one of the most substantial threats to geoheritage. Poorly managed geotourism can lead to site modification and degradation induced by tourists, accelerated weathering and erosion [23]. However, successful management strategies involving regulating visitor numbers, offering high-quality geoheritage experiences, implementing interpretative and appropriate infrastructure, and enforcing effective legislation can help mitigate the negative impacts of geotourism on geodiversity and heritage [21]. The dilemma between geotourism and geoconservation is a problematic point for geodiversity managers. Balancing the protection and exploitation of Geosites can be challenging [24], especially in developing countries where economic development resulting from geotourism is needed [21].

Another challenge for the management of geotourism is the difficulty of communicating understandable geological information to the public. The concept of Geoparks is still not well known by the public [25]. Thus, there is a low level of understanding and support from society and policymakers [13]. Insufficient funding for geoconservation, the absence of comprehensive geoconservation strategies, uncoordinated development of geotourism destinations and visitor management in the presence of natural hazards were also indicated as management challenges by Ólafsdóttir & Tverijonaite [21].

The intricacies of conserving geoheritage depend on various factors, including the type of heritage, climate, local customs and visitor behaviour [21]. The involvement of multiple stakeholders with differing goals, strategies and perceptions of geoheritage makes efficient management challenging, emphasizing the critical need for coordination among scientists, policymakers, planners, conservationists, tourism specialists, teachers and the public [24].

Despite the importance of geodiversity from both an ecosystem perspective and an economic perspective and all the challenges facing geological areas and Geoparks, very few studies focus on their conservation. Most studies in the literature focus on conserving biodiversity and ecosystem services. The economic valuation of biodiversity and ecosystem services is a common way of convincing policy makers of their importance. In this paper, we conduct a systematic literature review to identify gaps in the assessment of geological diversity to highlight areas where scientific contributions are needed to better protect geological resources. Thus, we are going to (i) identify the studies focusing on Geoparks and evaluate how they approach the issue of conservation of natural geoheritage and using which valuation techniques and (ii) highlight the current challenges faced when assessing Geoparks and geodiversity. This paper is organized as follows: in the second section, we explain the methodology used for the systematic literature review; in the third section, we present the results and a discussion of the systematic literature review; and lastly, in §4, we offer a brief conclusion.

## 2. Methodology

To select the papers to be included in our comprehensive systematic literature review, we followed three major steps: identification, screening and inclusion [26].

### (a) Identification of documents

In this step, Web of Science and SCOPUS databases were used to gather pertinent literature on the valuation of geodiversity and geological resources. To ensure the inclusivity of our search, we employed two to four search terms (keywords) for each query, resulting in a total of 120 search queries.

The chosen keywords were chosen to encompass both facets of the study, namely ‘the geological aspect' and ‘the valuation aspect'. The Boolean ‘AND' operator was used to create diverse queries by linking keywords from both aspects. For the geological aspect, terms such as ‘Geopark', ‘Geosite', ‘Geodiversity' and ‘Geoheritage' were used. Additionally, synonymous terms like ‘Geological diversity' for ‘Geodiversity' and ‘Abiotic ecosystem services' for ‘Geosystem services' were included using the Boolean ‘OR' operator. To capture the valuation aspect, terms like ‘Valuation' and ‘Assessment' were incorporated. Broadening our search, we integrated concepts such as ‘Total economic value' and ‘Cultural services'. We also included some of the most notorious assessment methodologies for valuing ecosystem services, such as ‘Choice experiment' and ‘Travel cost method', as keywords to further expand our search. Table 1 shows all search terms used for this study. The search period spanned from 26 March to 31 March, ensuring a comprehensive review of the available literature. The search terms were used on the title, abstract, and keywords and no date range was added.

**Table 1.** Search terms used in the systematic literature review.

search terms		
geological search terms	AND	valuation search terms
‘Geopark'		‘economic valuation'
‘Geoheritage' OR ‘Geological Heritage’		‘economic assessment'
‘Geodiversity' OR ‘Geological Diversity’		‘environmental valuation'
‘Geoconservation' OR ‘Geological Conservation’		‘environmental assessment'
‘Geosite' OR ‘Geological Site’		‘total economic value’
‘Geosystem Services’ OR ‘Abiotic Ecosystem Services’ OR ‘Geological Ecosystem Services’		‘use values’
		‘non-use values’
		‘option value'
		‘cultural services’
		‘supporting services’
		‘regulation services’
		‘provisioning services'
		‘travel cost method'
		‘hedonic price'
		‘contingent valuation method'
		‘choice experiment'
		‘choice modelling'
		‘willingness to pay'
		

The search queries generated a total of 204 documents, 74 from Web of Science and 130 from SCOPUS. This number was reduced to 61 after removing 127 duplicates and 16 documents to which we did not have access.

### (b) Screening of the documents

Based on our defined selection criteria, outlined in table 2, we proceeded to the screening of the abstracts, methodology sections and conclusions of the 61 documents. The initial screening phase targeted the identification of documents that were not papers, were not published in peer-reviewed journals or were not written in English. Following this step, three documents were excluded from our review: two papers in Spanish and one book chapter.

**Table 2.** Inclusion criteria of the documents to be included in the systematic literature review.

inclusion criteria	definition
type of the document	papers published in scientific peer-reviewed journals; books, book chapters and other documents should not be included
language	English; documents in other languages even if the abstract is in English should not be included
area of the study	Geopark, Geosites and specific geological areas; national parks and other parks and areas are not to be included unless the study is on a specific Geosite or geological area (aspect) within that park or area
objective of the study	the study needs to focus entirely or partly on an economic valuation of geodiversity or geosystem services

In the second screening phase, we focused on the study area and objective. In this phase, we excluded papers in which the study area was not a Geopark or a Geosite, and the objective of the study did not align with an economic assessment of geodiversity or geosystem services. The papers could be excluded based on one of the two criteria or both at the same time; for example, in the case of literature reviews. In total, 34 papers were excluded in this step.

### (c) Inclusion of the documents

**Figure 1.**
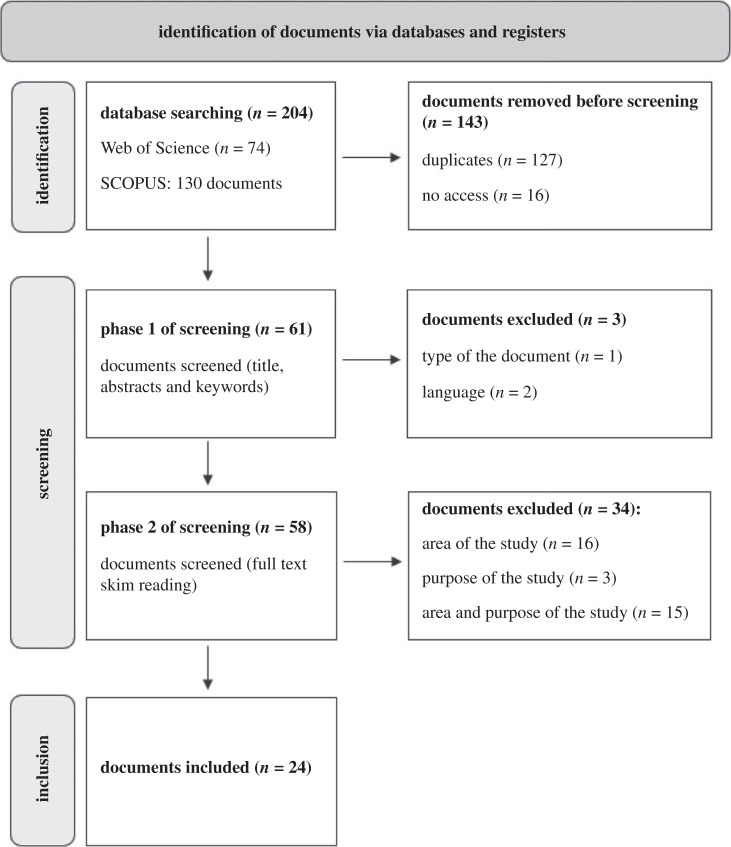
The chronological steps leading to the final selection of the reviewed documents.

In this step, 24 papers were fully read and analysed. Microsoft Excel was used to process the data. The chronological steps leading to the final selection of articles are visualized in figure 1.

An Excel table was created to systematically compile information from the papers examined in this literature review. Each entry in the table encapsulated essential details for comprehensive analysis, including the authors' names, publication year, country of the study, target population, designation of the study area, valuation techniques employed, study objectives, key findings, recommendations derived from the results, intended audience for the findings and a categorization of the geosystem services under consideration. This structured table facilitates a detailed and organized exploration of the diverse aspects covered in the selected papers.

## 3. Results and discussion

In this section, a detailed analysis is presented, delving into key aspects within the selected papers for our systematic literature review. Our examination puts particular emphasis on the temporal and geographical dimensions of the reviewed papers, the employed valuation techniques and overarching objectives, the economic values placed on Geoparks and Geosites as well as the threats facing geodiversity and affecting the management of these resources.

### (a) General overview of the years and areas of study of the reviewed papers

The examined studies cover a period from 2012 to 2023, indicating a recent interest in the valuation of geodiversity. Table 3 provides a chronological overview of these studies, highlighting a notable surge in research activity since 2012. The first study in a peer-reviewed journal with an economic valuation of geological resources in English was only published in 2012. This trend suggests an increasing focus on this field, possibly driven by the emerging prominence of geodiversity and Geoparks as research subjects [1]. The term ‘geodiversity' was introduced in 1993 after the Rio Earth Summit in 1992. However, the term ‘geosystem services' is a relatively newer concept and is also associated with the broader idea of ecosystem services. The lack of awareness and understanding among policymakers, researchers, and the general public may be behind the delayed interest in the assessment and then conservation of geosystem services.

**Figure 2.**
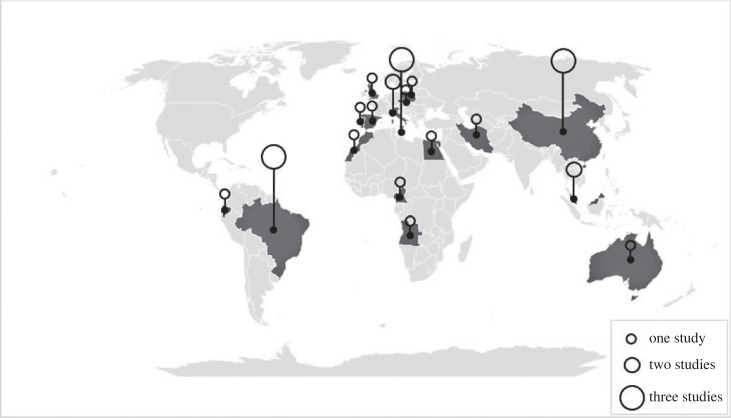
Geographical representation per country of the reviewed papers.

**Table 3.** Overview of the years of the reviewed papers.

year	no. studies	authors
2012	1	Coratza *et al*.
2013	2	Špaček & Antouškova; Pereira *et al*.
2014	2	Tefegoum *et al*.; Cheung *et al*.
2016	3	Cheung; Perez-Alvares *et al*.; Najwer *et al*.
2017	1	Bouzekraoui *et al*.
2018	3	Rahman *et al*.; Cappadonia *et al*.; Martins & Pereira
2019	3	Selmi *et al*.; Lopes *et al*.; Rangel *et al*.
2021	4	Nascimento *et al*.; Ferrando *et al*.; Williams & McHenry; Carrión-Mero *et al*.
2022	3	Martino *et al*.; Mehni *et al*.; Zhu
2023	2	Dani *et al*.; Ahmed

Despite the limited number of studies, our analysis shows a broad geographical representation; economic assessments of geological resources have been conducted in five out seven continents (figure 2). The investigated Geosites and Geoparks by the reviewed papers are primarily situated in Europe with a total of nine studies, including two studies in Malta [27,28], and individual studies in Italy [29], Spain [30], Poland [31], Czech Republic [32], Portugal [33] and the UK [34]. Additionally, one study spans two countries, Malta and Italy [35]. Following Europe, Asia accounts for a total of six studies, with three conducted in China [36–38], two in Malaysia [39,40] and one in Iran [41].

The large number of papers in Europe and Asia, compared to the other continents, is also shown by the bibliometric analysis of the Geopark research by Herrera-Franco *et al*. [22]. According to Herrera-Franco *et al*. [22], China emerges as the leading contributor in the field of Geoparks, calculated by the number of scientific publications, and is followed by other countries in Asia and Europe. This concentration can be explained by the fact that most Geoparks are primarily concentrated in Europe and Asia and the slightly heightened awareness of geodiversity's significance in these areas.

Africa and South America come in the third place with four studies each. In Africa, one study is conducted in each of Cameroon [42], Morocco [43], Angola [44] and Egypt [45]. In South America, three studies take place in Brazil [46–48], and one in Ecuador [49]. Australia, representing the final continent, accounts for only one study [50].

**Figure 3.**
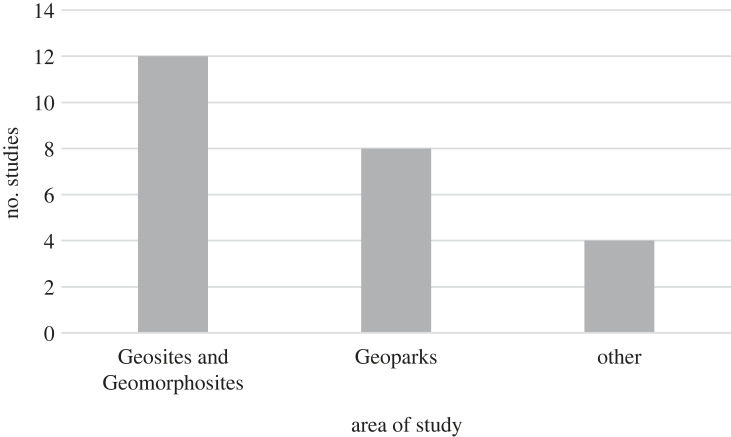
Overview of the type of the study areas in the reviewed papers.

The majority of the studies, as shown in figure 3, conduct valuations on either Geosites [28–30,35,44,45,48,49] or geomorphological sites [27,33,42,43]. The scope of these valuations varies, ranging from a few sites in certain studies (e.g. Carrión-Mero [49] assessed geodiversity on four Geosites) to a more substantial number in others (e.g. Ferrando *et al*. [29] examined 120 Geosites). Geosites are defined as ‘areas of special geological and geomorphological significance' [51], while Geomorphosites are Geosites that specifically emphasize landforms and the processes contributing to their formation, encompassing a broader range of geological features [52].

The second most prevalent study area in the selected papers is Geoparks [32–34,36,41,49]. Geoparks are defined as ‘areas where a network of Geosites is maintained, serving as the main locations for visits and educational activities' [51]. Several studies are conducted within specific Geoparks located in Europe and Asia, such as the Hong Kong UNESCO Global Geopark in China, North Pennines AONB UNESCO Global Geopark in the UK, Langkawi UNESCO Global Geopark and Kinabalu UNESCO Global Geopark in Malaysia, and Bohemian Paradise UNESCO Global Geopark in the Czech Republic. Other studies explored potential Geoparks, such as Hormuz Island in Iran.

Three studies did not focus on specific Geoparks or Geosites [31,46,47,50]. Rangel *et al*. [47], for instance, examined geodiversity values within a national and state park.

### (b) Valuation approaches and objectives

Multiple approaches have been employed in the selected papers to assess geodiversity. Table 4 offers a comprehensive overview of the diverse valuation methodologies applied in the assessment of geodiversity and geosystem services across the examined papers. The Geosites assessment methodologies (GAM) emerges as the predominant approach with a total of 10 studies. GAM is a structured approach employed to systematically evaluate geological sites, encompassing scientific, additional (aesthetic and cultural) use and degradation risk values. This approach, originally formulated by Brilha *et al*. [53], Brilha [54] and Vujicic *et al*. [55], underwent multiple improvements over the years. The most recent one was by Williams & McHenry [50] through the integration of digital tools.

**Table 4.** Overview of the valuation techniques employed in the reviewed papers.

valuation technique	type of technique	no. studies	authors
Geosite assessment methodologies	qualitative	10	Coratza *et al*. [27]; Pereira *et al*. [46]; Bouzekraoui [43]; Martins & Pereira [33]; Lopes *et al*. [44]; Selmi *et al*. [28]; Williams & McHenry [50]; Nascimento *et al*. [48]; Ferrando *et al*. [29]; Ahmed [45]
contingent valuation method	quantitative	5	Cheung *et al*. [36]; Cheung [37]; Rahman *et al*. [39]; Mehni *et al*. [41]; Dani *et al*. [40]
ranking-based assessments	qualitative	4	Tefegoum *et al*. [42]; Najwer *et al*. [31]; Cappadonia *et al*. [35]; Rangel *et al*. [47]
travel cost method	quantitative	2	Špaček & Antouškova [32]; Perez-Alvares *et al*. [30]
SolVES model	qualitative	1	Zhu [38]
cause–effect method and tourist carrying capacity	qualitative	1	Carrión-Mero [49]
choice experiment	quantitative	1	Martino [34]

Despite GAM's prevalence, some limitations have been identified, primarily its exclusive reliance on scientific expert viewpoints. In recognition of the importance of qualitative dimensions, certain studies have incorporated people's perceptions into the valuation process. For instance, Ahmed [45] and Martins & Pereira [33] integrated the qualitative aspect by considering the perspectives of visitors and inhabitants, respectively.

The contingent valuation method (CVM) emerges as the second most employed approach with five studies. CVM, a survey-based economic method, is used for estimating the economic value of non-market goods and services, especially those lacking readily observable market prices. This method entails presenting individuals with hypothetical scenarios, prompting them to express their willingness to pay (WTP) or accept compensation (WTA) for a specific environmental resource. In the context of geodiversity and geosystem services, CVM was employed to assess the economic value individuals place on these non-market aspects of the environment. Surveys presented respondents with scenarios outlining potential impacts on geodiversity or geosystem services, eliciting their monetary valuation. A parallel methodology to CVM is the choice experiment (CE), also a survey-based approach estimating the economic value of non-market goods and services by presenting respondents with hypothetical scenarios. The key difference lies in CE defining the services or goods to be assessed using different characteristics. Despite being more intricate than CVM, CE proves more suitable for evaluating geosystem services due to their complex nature. CE accommodates complex goods or services with multiple attributes, providing flexibility and efficiency in the valuation process. Within our systematic literature review, one paper used this methodology, leaving opportunities for further studies to emerge [34].

Ranking-based assessments represent the third most prevalent method in the reviewed papers, with four studies, followed by the travel cost method (TCM). Ranking systems are employed for qualitative assessments of geodiversity and geosystem services, often based on Gray's [56] procedure [42,47]. Najwer *et al*. [31] used the ranking-based assessments to create seven map factors which became the basis for the creation of total geodiversity and biodiversity maps. On the other hand, TCM is an economic valuation technique specifically designed to estimate the economic value of non-market goods and services, particularly in recreational and natural environments. It assesses the value individuals assign to a site by analysing the costs incurred for travelling to and enjoying that location. TCM is well suited for evaluating the economic benefits associated with natural and recreational areas, making it a valuable method for assessing the value of geodiversity from a visitor's perspective. However, it is noteworthy that only two papers in the systematic literature review used this method [30,32].

Additionally, other techniques like SolVES model, cause–effect method and tourist carrying capacity (TCC), applied in individual studies, present further avenues for exploration in future research. SolVES is a model used to assess the social values of ecological services [38]. It is an application based on geographical information system technology that quantifies various social values of ecosystem services and conducts spatial analysis. The model's spatial analysis module comprises social value, value mapping and value conversion mapping. It aims to incorporate the social dimension into ecological services assessments by considering people's perceptions and values associated with different landscapes, enabling informed decisions about land use, conservation and resource management. Despite being a powerful tool, this model has some limitations, primarily simplifying complex human–environment interactions and lacking the ability to capture dynamic changes in social values over time.

The cause–effect method is an interactive matrix implemented for environmental impact studies to illustrate the interactions between two components: natural and anthropogenic. This matrix is a double-entry table where the rows represent the environmental factors to be assessed, and the columns contain the values corresponding to each factor. Carrión-Mero [49] combines this method with the TCC, which involves three components: the physical carrying capacity (PCC), real carrying capacity (RCC) and effective carrying capacity (ECC). The PCC denotes the limit of visits that can be made to the site per day, while the RCC includes a series of correction factors to the PCC that affect the site directly or indirectly. Finally, the ECC is defined as the maximum number of visits that can be allowed, considering the management capacity. The combination of the cause–effect method and the TCC by Carrión-Mero [49] involves three steps: (i) strategic Geosite selection; (ii) environmental assessment of Geosites and analysis of their carrying capacity for tourism; and (iii) interpretation of results and strategy development. The cause–effect method was used for the environmental assessment, and the TCC was implemented to calculate the physical carrying capacity of the Geosites. The primary limitation of this approach is its failure to adequately incorporate the perspectives of local communities, tourists, or other stakeholders, potentially leading to a lack of consideration for their values and concerns.

The main objective of the reviewed papers is to assess both the monetary and non-monetary value associated with specific Geoparks, Geosites or geodiversity features, providing insights for policymakers and decision-makers, particularly in the context of geoconservation. The papers can be categorized into three groups based on their specific objectives.

Firstly, some papers focus on estimating the value attributed by visitors to recreational activities within Geoparks or the overall recreational value of the Geopark. Their aim is to comprehend and underscore the role of recreational activities in attracting visitors to Geoparks and Geosites. Secondly, other studies concentrate on identifying the value that visitors place on conserving geological elements, providing an additional argument for preserving geosystem services. Lastly, there are papers dedicated to assessing the geoheritage at Geosites or geomorphological sites, relying on expert opinions and literature reviews to evaluate the geological potential of these sites, considering their suitability for educational purposes and geotourism.

The studies in the first and third categories emphasize the recreational value of Geoparks, ultimately promoting geotourism, and hence, bringing more attention, revenue and support for Geoparks' maintenance. However, increased visitation, beyond a certain threshold, can impact geological resources and the overall ecosystem. In the second category, studies focus on quantifying the value visitors place on conserving geological elements, providing an additional argument for geoconservation. The synergy between these categories can be a starting point to establish a harmonious balance where geotourism becomes a positive force for conservation efforts. However, achieving this synergy requires further research. More studies are needed to balance the number of papers in each category. In this context, future research should focus on determining the equilibrium point of geotourism and conservation efforts, with studies like Carrión-Mero [49] exploring tourist carrying capacity to enhance sustainable Geosite use.

The majority of the selected scientific papers (13 studies) do not employ surveys and hence do not target any specific group (table 5). Nine studies targeted park visitors. Typically, these papers surveyed a broad spectrum of tourists through questionnaire surveys, with the exception of Špaček & Antouškova [32], who exclusively focus on domestic tourists and Rahman *et al*. [39], who enhance their survey with observations and complementary interviews.

**Table 5.** Overview of the target groups in the reviewed papers.

target groups	no. studies	authors
no survey	13	Coratza *et al*. [27]; Pereira *et al*. [46]; Tefegoum *et al*. [42]; Najwer *et al*. [31]; Bouzekraoui [43]; Cappadonia *et al*. [35]; Selmi *et al*. [28]; Lopes *et al*. [44]; Rangel *et al*. [47]; Ferrando *et al*. [29]; Williams & McHenry [50]; Nascimento *et al*. [48]; Carrión-Mero [48]
visitors	9	Špaček & Antouškova [32]; Cheung *et al*. [36]; Cheung [37]; Perez-Alvares *et al*. [30]; Rahman *et al*. [39]; Mehni *et al*. [41]; Martino [34]; Ahmed [45]; Dani *et al*. [40]
experts	1	Zhu [38]
inhabitants	1	Martins & Pereira [33]

Only one study focuses on local inhabitants as the target population [33]. Through questionnaires, this study engaged inhabitants to gather insights into their perceptions of various values associated with geomorphosites. The research aims to emphasize the importance of educating local residents about the value of geomorphosites in their daily surroundings, encouraging them to actively preserve their cultural heritage. Since inhabitants of Geoparks and Geosites are in direct contact with geosystem services, they can either contribute to their destruction or aid in their conservation. Thus, conducting studies targeting inhabitants, examining their behaviour, perceptions and preferences, can significantly contribute to geoconservation efforts.

### (c) The monetary value of Geoparks, Geosites or geoconservation

The majority of the reviewed papers employ qualitative valuation techniques (table 5). Some of these studies assigned values to Geosites using ranking or scoring systems without providing a monetary assessment, as observed in the works of Coratza *et al*. [27] and Williams & McHenry [50]. These qualitative studies often involve the creation of inventories of geosystem services. However, Williams & McHenry [50] highlight challenges faced by researchers attempting to evaluate geodiversity, citing issues such as scale, logistical challenges in boundary detection and assessment, and financial restrictions for accessing challenging terrain.

By contrast, fewer studies (33%) conducted quantitative research, offering insights into the monetary value associated with Geoparks, Geosites or geoconservation. Using the CVM, Cheung *et al*. [36] estimate the average willingness to pay for Geopark management and conservation at HK$134.90 (equivalent to €15.68) per visitor. Perez-Alvares *et al*. [30] calculate the total economic value of a Geosite which amounts to €34 961 162. Cheung [37] discovers that visitors are willing to pay HK$165.30 (equivalent to €19.22) for an accredited tour showcasing geodiversity. Rahman *et al*. [39] find that respondents are willing to contribute between RM50 (equivalent to €9.74) and RM200 (equivalent to €38.97) to landscape conservation. Mehni *et al*. [41] estimate the total annual recreation value of the Geopark for visitors at $38.84 (equivalent to €35.24) per hectare, while Dani *et al*. [40] indicate that respondents are willing to pay a higher admission fee for more significant geoconservation, resulting in a willingness to pay of RM4.98 (equivalent to €0.97). Through TCM, Špaček & Antouškova [32] calculate the impact of recreational geosystem services on the consumer surplus (CZK497.90 equivalent to €20.23). The study emphasizes that most tourists travel between 61 and 90 min to reach the Geopark, with around half of the visitors spending CZK50 to 100 (equivalent to €2.03 to €4.06) on transportation costs.

These quantitative studies offer a practical and measurable approach to understanding the economic dimensions of nature, gauging visitors' willingness to pay for specific experiences, conservation efforts, or recreational activities within geological areas. However, a broader discussion has unfolded regarding the monetization of nature, especially in the context of ecosystem services. The act of ‘economizing' nature involves assigning economic value to these services, aiming to integrate them into economic decision-making. While this approach is promising as it acknowledges nature's importance and promotes responsible resource management, critics argue that reducing nature to economic terms risks instrumentalizing and commodifying natural resources within existing capitalist structures. This dilemma underscores the challenge of integrating ecological considerations into economic frameworks without reinforcing the structures contributing to environmental degradation. Critics emphasize the tension between ‘economizing' nature and challenging the status quo of capitalism.

In our context, assigning economic value to geosystem services may enhance geoconservation efforts but it could also co-opt ecological concerns into a profit-centric system. Addressing this, in the context of geoconservation, involves questioning the fundamental principles of capitalism and exploring alternative economic models prioritizing ecological well-being alongside human welfare.

### (d) Threats to geodiversity and management issues

In our analysis, we examined the extent to which threats to geodiversity and management issues of Geoparks or Geosites were addressed in the studies. One of the most significant threats to Geoparks and geodiversity is the impact of tourism on the environment and geological resources [21]. This concern was echoed by several of the analysed studies. Cheung [37] points out that the rapid growth of visitors in a park can have severe and irreversible impacts on the natural environment, with improper tourist behaviour negatively affecting the area's geological landscape. Lopes *et al*. [44] note that while tourism and leisure activities can contribute to societal and economic development, they can pose significant threats if not managed properly. Coratza *et al*. [27] emphasize that while tourism in Malta is one of the most important economic activities, it negatively affects coastal resources, leading to marine and air pollution, loss of natural habitats, land degradation and urbanization. Selmi *et al*. [28] further highlight the problem of a high annual number of tourists and crowding as a significant threat to Geosites. To mitigate these risks, sustainable management strategies should be implemented within a framework of geoconservation to preserve the existing geoheritage. Rangel *et al*. [47] provide an example of the damaging consequences of poor management, highlighting that due to inadequate planning and management, the trails used by both visitors and local people to access geotourism locations are in bad shape. Such mismanagement can lead to soil degradation, a decrease in soil quality and its capacity as an environmental regulator if not considered in time.

The balance between geotourism and geoconservation, often referred to as the protection versus development dilemma, is a central theme in many of the reviewed papers. Martino [34] notes a potential value conflict between the benefits of conservation and the preferences of certain recreationists. Rahman *et al*. [39] observe that the village landscape they study is under pressure to be transformed for socio-economic needs and rapid urbanization, resulting in the erosion of the natural environment. Cappadocia *et al*. [35] argue that the priority for geotourism is to preserve the landscape, allowing development while respecting the natural environment based on sustainability principles.

Despite these papers addressing the importance of the balance between conservation and tourism, and human activity in general, on geological landscapes, no practical solutions are provided. The issue remains a hot topic, and more research and funds should be invested in tailoring management strategies to help find this delicate balance. Management strategies should be well-targeted and context-fitting, as a universal solution might not be the most effective approach. Despite the already broad geographical representation of the selected papers, the expansion of the geographical scope of research in the future is recommended.

Threats to geological resources are not solely limited to human activities; natural processes can also compromise their value. Therefore, the conservation of these resources must address the problem of both the destruction caused by natural and active processes, and man-made damage, making geoconservation a very complex issue [28]. Nascimento *et al*. [48] emphasize the need for enhanced protection of geological landscapes with a high or moderate risk of degradation due to natural or anthropic processes. These areas are often located in potentially degrading zones, such as mining, urban areas, industrial facilities, recreational areas, and road and rail structures. The risk of degradation is also heightened in the absence of protection measures or access control, particularly in easily accessible areas with high population density.

The analysis of the selected papers revealed additional challenges that have been acknowledged by authors. Studies by Mehni *et al*. [41] and Williams & McHenry [50] underline the critical issue of insufficient financial resources, particularly prevalent in underdeveloped countries. This financial constraint poses a significant barrier to the effective management and conservation of Geosites. Climate change, as highlighted by Selmi *et al*. [28], introduces an additional layer of threat to Geosites. Alterations in climate patterns can induce changes in geological features and processes, thereby impacting the overall integrity of these sites. Martins & Pereira [33] draw attention to the risk of neglecting local communities. Actively involving the local population and addressing their perspectives and concerns are vital components to garner support for geoconservation initiatives. Williams & McHenry [50] identify challenges related to the limited understanding of geoheritage and geotourism. This encompasses issues such as insufficient promotion and awareness, potentially leading to a lack of interest and engagement from both local and global communities. Furthermore, Williams & McHenry [50] stress the importance of addressing problems related to land tenure and legal protection. The absence of clear legal safeguards and land tenure can expose Geosites to various threats, including inappropriate land use and development. Nascimento *et al*. [48] emphasize the pivotal role of raising awareness among local communities. Recognizing the significance of Geosites and fostering collective efforts in geoconservation can contribute substantially to their sustainable preservation. While these issues have been acknowledged in the selected studies, further exploration is imperative for the effective conservation and sustainable management of Geosites on a global scale.

## 4. Conclusion and recommendations

Overall, this paper highlights the important gap in the literature regarding the assessment of geological resources and their diversity. However, assessing these resources and their diversity is crucial to inform on the best management options, allowing policymakers to prioritize the high risk of degradation and focus their efforts accordingly. Thus, focusing on the valuation of geological resources in future research is mandatory for their conservation.

The current literature predominantly relies on GAM and CVM to assess the quantitative and qualitative values of geodiversity and geological resources. Despite exploring alternatives like TCM and CE, there is a compelling need to develop holistic methodologies integrating scientific, economic and qualitative perspectives for a comprehensive valuation of geodiversity and geosystem services. Future research should aim at developing innovative approaches that bridge these gaps and contribute to a more holistic understanding of the value of geological resources.

Most of the reviewed papers are policy targeted. Their main objective is often to help make more efficient management structures and strategies to conserve geological services and related geoheritage. However, they are primarily focused on visitors' perspectives of evaluation. It is crucial for future studies to prioritize the perspectives of local inhabitants, acknowledging their unique viewpoints and contributions to the understanding and conservation of geological resources.

These papers also show visitors' crucial role in developing the local economy of geological areas through geotourism. This highlights the dilemma that most geological sites and parks struggle with: ‘conservation versus development and economic growth'. New research should focus more on finding how to balance between the protection and conservation of geodiversity, and developing sustainably geological areas to promote geotourism.

In conclusion, geodiversity lays the foundation for geosystem services, serving as the geological and environmental underpinning for the goods and functions associated with landscapes. This conceptual framework underscores the crucial role of abiotic elements in the intricate balance of ecosystems, mirroring the role played by biodiversity in supporting ecosystem services. The seminal works of Gray [1,2] and Kozłowski [3] underpin these conceptualizations, emphasizing the evolving importance of integrating geodiversity and geosystem services into contemporary environmental discourse. Though recognizing the importance of the ‘geosystem services' framework, it is important to acknowledge that these services, by their nature, reflect anthropocentric and utilitarian perspectives, concentrating on the benefits these systems provide to human well-being (visitors and inhabitants). This approach inherently places human interests at the centre, potentially undervaluing the intrinsic value of ecosystems independent of their utility to humans. While acknowledging the practical necessity of assessing these services for human welfare, it is crucial to recognize and address the limitations of an anthropocentric lens in future research.

## Data Availability

This article has no additional data.

## Appendix A. List of articles included in the systematic literature review

See references [27–50].
